# How culture affects the way in which psychopathologies manifest in behavior: The case of Confucianism in China

**DOI:** 10.1177/00207640241262716

**Published:** 2024-07-24

**Authors:** Lorenza Colzato, Liang Zhang, Wenxin Zhang, Christian Beste, Bernhard Hommel, Veit Roessner

**Affiliations:** 1 Department of Psychology, Shandong Normal University, Jinan, China; 2 Cognitive Neurophysiology, Department of Child and Adolescent Psychiatry, Faculty of Medicine, TU Dresden, Dresden, Germany

**Keywords:** externalizing psychopathologies, internalizing psychopathologies, Confucianism, culture, China

## Abstract

**Background:**

Internalizing disorders in children and adolescents are about as frequent as externalizing disorders in the US, but three times more prevalent than externalizing disorders in China.

**Aims:**

To examine why and how mental predispositions and stress lead to psychopathology in general and manifest as internalizing or externalizing problems in particular, and which role intercultural differences may play in understanding this.

**Method:**

A review of the literature.

**Results/conclusions:**

The interplay of personal freedom and societal duties in an individual’s development crucially influences whether psychopathologies appear as externalizing or internalizing issues. Eastern and especially Chinese cultures have long favored societal obligations over individual autonomy, guided by Confucian principles, promoting internalization over externalization. Understanding culture’s role in behavior can improve mental healthcare by fostering tailored, culturally informed interventions for children and adolescents.

Poor mental health in adolescence has become a major issue worldwide, especially in terms of depression (Shorey et al., 2022) and anxiety (Solmi et al., 2022). However, even though the overall incidence rates are commonly comparable across countries, the way psychopathological problems translate into overt behavior shows remarkable differences between Eastern (Asia and the Middle East) and Western countries (South and North America, European countries, New Zealand, and Australia). Various psychopathologies can translate into so-called internalizing and externalizing behaviors (Achenbach et al., 2016). Whereas internalizing consists in dysfunctional behavior directed inward, such as anxiety, depression, obsessions, compulsion, withdrawal, and suicidal tendencies, externalizing consists in dysfunctional behavior directed outward (toward the environment or other people), such as aggression, impulsivity, opposition, and substance use (Achenbach et al., 2016). So far, it is insufficiently understood why and how mental predispositions and stress lead to psychopathology in general and manifest as internalizing or externalizing problems in particular. Interestingly, internalizing disorders in children and adolescents is about as frequent as externalizing in the US (Merikangas et al., 2010), but three times more prevalent than externalizing disorders in China (Xiaoli et al., 2014). Although differences in overall prevalence may reflect different ways of assessment, diagnostic procedures, thresholds, and other characteristics of the respective mental health systems, these drastic differences in prevalence ratios of externalizing to internalizing disorders are unlikely to be due to any of these factors. Here we argue that the consideration of intercultural differences might provide a better understanding of this discrepancy.

More specifically, we suggest that the culturally motivated balance between personal autonomy (individualism) and social responsibility (collectivism) of the developing individual plays a decisive role in channeling the manifestation of psychopathologies as either externalizing or internalizing behaviors, or even disorders. One of the key differences between many Western and Eastern countries and cultures is the degree of individualism versus collectivism. In Western countries, children and adolescents are taught and expected to prioritize individual goals, achievements, and success over collective goals and responsibilities. Children and adolescents are strongly encouraged to pursue their self-interests and aspirations, and competition is promoted as a means of achieving personal success. This can lead to a lack of concern for the well-being of others and a reduced sense of social responsibility. Therefore, individuals should be more likely to channel mental stress into behaviors that benefit themselves at the expense of others, such as aggression or rule-breaking behaviors. Furthermore, in individualistic societies, children and adolescents often have greater freedom and autonomy in their decisions and actions, which can lead to a sense of entitlement and a lack of accountability for their behavior, thus increasing the likelihood of externalizing psychopathological problems.

Many Eastern countries, and China in particular, have cultural traditions that promote a balance between autonomy and social responsibilities that is much more biased toward the latter. China’s approach to this balance is best expressed by Confucian thinking, which has shaped the country’s philosophical attitude for more than 2,000 years and which, after a few decades of official disapproval during the reign of Mao Zedong, has regained full support by government and people (Pang, 2019). While Confucianism may also have a positive impact on mental health (Wang et al., 2016), it can be assumed to strongly promote internalization over externalization. This is for at least three reasons: First, Confucianism has a negative view on mental illness, which may lead to stigmatization and reluctance to seek psychological help (Yu et al., 2018). It stresses the cultivation of virtue and moral character and assigns moral responsibility to learn, grow, and strive toward moral perfection to the person. Accordingly, mental illness is perceived as personal failing or weakness in character. Second, Confucianism emphasizes harmony in societal and familial relationships, which is assumed to be disturbed by mental illness (Li et al., 2022), which in turn is likely to cause shame and guilt in individuals suffering from mental health issues.

Third, while it is widely acknowledged that China has a strongly collectivistic society, the fact that it is vertical, rather than horizontal collectivism in the sense of Hofstede (Minkov & Hofstede, 2011) has often been ignored. While horizontal collectivism emphasizes equity and goal-sharing (like in Kenya, which relies on the cultural influence of Harambee; Vershinina et al., 2017), vertical collectivism relies on hierarchy and social order within a group. This view is strongly emphasized in Confucianism, which promotes respect for authority, obedience, and loyalty to the group. Decision-making is often centralized, with power and resources concentrated on a small elite, which often acts like a head of a family, rather than elites and decision makers in typical Western societies. Given these characteristics, we do not suggest that internalizing problems are more prominent than externalizing problems in all collectivist societies. Rather, it may be the specific combination of verticality and collectivism, as propagated by Confucianism, that renders internalization more likely than externalization (Lansford et al., 2018).

Our argumentation calls for more consideration of individual differences rooted in the cultural values that are driving the way young people are brought up. The tradition of Confucianism in China may be one example, and the Weberian combination of Protestant ethic and spirit of capitalism (Davies, 1992) may be another. This implies, among other things, that conventional treatment models, which are primarily influenced by Western psychiatry and tailored toward patients from WEIRD (Jacobs et al., 2015) countries, may not align with the needs and expectations of individuals from countries with another cultural background. By considering the influence of cultural values, mental health professionals can adapt and develop therapeutic approaches to deal with concepts like social harmony and respect for authority, say, so to ensure that treatments are culturally sensitive and more likely to be effective. By recognizing and studying the unique manifestations of externalizing or internalizing problems influenced by culture, we can move away from a one-size-fits-all approach to mental healthcare by fostering more tailored, culturally-informed and thus more effective treatment strategies. Such strategies could include cultural competence training to enhance the cultural awareness of mental health professionals, cultural assessment of the individuals seeking mental health support, the provision of culturally sensitive environment within mental health facilities and the collaboration between mental health professionals and community leaders, cultural experts, and interpreters. This shift might promote a more nuanced understanding and better diagnosing of mental health, and encourage the development of tailored interventions that respect and incorporate cultural values to a degree that will likely increase the mental health of all children and adolescents.
